# Measuring Scholarly Productivity: A Primer for Junior Faculty. Part III: Understanding Publication Metrics

**DOI:** 10.5811/westjem.2018.9.38213

**Published:** 2018-10-18

**Authors:** Linda S. Murphy, Chadd K. Kraus, Shahram Lotfipour, Michael Gottlieb, James R. Langabeer, Mark I. Langdorf

**Affiliations:** * University of California-Irvine Libraries, Reference Department, Irvine, California; † Geisinger Health System, Department of Emergency Medicine, Danville, Pennsylvania; ‡ University of California Irvine Health School of Medicine, Department of Emergency Medicine, Irvine, California; § Rush University Medical Center, Department of Emergency Medicine, Chicago, Illinois; ¶ University of Texas McGovern School of Medicine, Houston, Texas

## Abstract

There are approximately 78 indexed journals in the specialty of emergency medicine (EM), making it challenging to determine which is the best option for junior faculty. This paper is the final component of a three-part series focused on guiding junior faculty to enhance their scholarly productivity. As an EM junior faculty’s research career advances, the bibliometric tools and resources detailed in this paper should be considered when developing a publication submission strategy. The tenure and promotion decision process in many universities relies at least in part on these types of bibliometrics. This paper provides an understanding of new, alternative metrics that can be used to promote scientific progress in a transparent and timely manner.

## INTRODUCTION

Understanding the strength and weaknesses of different publication metrics and deciding where to publish your research is crucial in today’s competitive academic environment. Publishing papers in quality journals provides the best method to disseminate your work and increase your research exposure.

There are approximately 78 indexed journals in the specialty of emergency medicine (EM). While you can choose to submit your paper to any of these journals, it can be challenging to determine the best option for your research needs. This paper is the last of a three-part series focused on guiding junior faculty to enhance their scholarly productivity.^1^,^2^ The first paper discussed strategies for effective writing and publication.^2^ The second paper^1^ highlighted promotion processes in one’s career. This last paper provides an in-depth narrative review of different publication metrics that are used to measure the impact of published research.

Understanding the complexity of various bibliometric tools and their parameters can be a challenge. This paper will discuss the traditional metrics in the context of journal, article, and author level in addition to the rising importance of alternative metrics. Our goal is to provide junior researchers with a primer on how these metrics are calculated, as well as their benefits and pitfalls. We will then offer strategies for incorporating these to maximize your academic success: suggestions on journal selection, methods to track your research impact for academic achievement and potential collaborative work, and finally, tips on how to detect misleading metrics and impact factors that are not widely accepted in the scientific community.

### Bibliometrics: Why it matters?

Bibliometrics is the quantitative analysis of scholarly publications. It quantifies both the quality and research impact of an author’s productivity, and the prestige of a journal.^3^ Citation analysis measures the impact of both a journal and an author’s research impact. It generates the number of publications by an author, the total citations received from these publications, and the prestige of the journals in which those articles were published.^4^

### Journal-Level Metrics

There are several journal-level metric tools, but the data are mostly generated from two major indexing databases: Web of Science (WOS) and Scopus. Both databases allow users to search articles on a topic, track scholarly impact of a journal or individual author, and retrieve a list of journals in a specific field, e.g., journals in EM.

#### Journal Impact Factor and the Journal Citation Report

The WOS Core Collection is a multidisciplinary database provided by Clarivate Analytics (formerly ISI Thomson Reuters) that indexes over 20,300 journals in the Science Citation Index Expanded (SCIE), Social Sciences Citation Index (SSCI), and Arts & Humanities Citation Index (HCI). The Core Collection also provides the journal impact metrics found in Journal Citation Reports (JCR).^5^ For decades, the Journal Impact Factor (JIF) has been the primary metric to evaluate the citation frequency of a scientific journal.^4^,^6^ Published annually since 1975, JIF has long been the gold standard for librarians, researchers, and decision-makers to compare peer-review journals and research impact within a specific field.^7^ Librarians use JIF as a criterion for journal selections, authors use it for deciding where to publish, academic officials use it for recruitment and promotion, and funding agencies use it for grant allocation.^8^ JIF is a measure of the average frequency with which articles in a journal are cited. The data are gathered in WOS JCR that lists journals and their impact factors. The journals are categorized and ranked in the context of their specific field(s). The “two-year” JIF, though an arbitrary regarding time, is the most widely considered, as it provides a moderate period for other authors in the field to take note of, and reference the work. The method of calculation for an example two-year JIF 2017 is described below:

$$\text{Year }2017\;\text{JIF}=\frac{\text{Citations received to items published in }2015+2016}{\text{Number of substantive articles }(\text{i.e.},\;\text{exclude editorials and letters})\;\text{published in }2015+2016^{4,6}}$$

##### The Limitations of JIF

- In addition to the narrow two-year window metric calculation, the journal indexing coverage in SCIE is limited to 1,090 journals (http://mjl.clarivate.com/cgi-bin/jrnlst/jlresults.cgi?PC=D). Less than 30 EM journals were categorized, indexed, and reported in the 2017 JCR.
- The influence of self-citation boosts the impact factor and only citable articles are included.^9^
- It does not discriminate between higher and lower quality articles published in the journal.^10^,^11^ It only counts the number of citations received and ignores information about those citation sources.
- JIF is biased toward certain fields of research; EM is a relatively new medical specialty. (The specialty’s first journal, *Journal of the American College of Emergency Physicians*, was first published in 1972 and later renamed *Annals of Emergency Medicine*).^12^ EM journals generally rank lower in impact factor among specialties.^13^ For instance, the median impact factor found in the 2017 JCR report for the 26 EM journals was 1.391 as comparing to 3.186 for 222 journals in oncology.^14^
- JCR is a fee-based, expensive resource that is mostly subscribed to by major academic libraries.

#### Eigenfactor vs. Journal Impact Factor

The Eigenfactor algorithm uses citation data from JCR to assess and track the influence of a journal in relation to other journals.^15^,^16^ The Eigenfactor measures the journal’s overall importance by counting the total number of citations a journal receives over a five-year period. As a result, a journal that publishes a large number of articles is more likely to have a higher Eigenfactor Score (ES). Examples for this scenario are shown in Table 1. *Am J Emerg Med* was ranked #4 in Eigenfactor, but was weighted much less in JCR (#21), SJR (#20), and CiteScore (#28). As opposed to the journal *Emergencias*, which was ranked #4 by JCR, but was weighted outside the top 20 EM journals by Eigenfactor (ES = 0.00116), and was ranked within the 74-50th percentiles (second quartile) by Scopus and SJR (https://www.scimagojr.com/journalrank.php?area=2700&category=2711).

The impact factor measures citations per article, and can be a useful metric tool for authors when choosing a journal to submit their manuscripts. Eigenfactor, on the other hand, measures a journal’s overall importance and the influence in its scientific community. The data are used by librarians in supporting their journal selection, decision-making process.^16^ The Eigenfactor.org website provides a free searchable database of journal ranking (http://www.eigenfactor.org/projects/journalRank/journalsearch.php). By selecting “*Year, 2015*” and “*Emergency Medicine & Critical Care*” as the ISI Category, you will retrieve the Eigenfactor journal ranking of the 24 EM Journals from JCR (http://www.eigenfactor.org/projects/journalRank/rankings.php?search=FF&year=2015&searchby=isicat&orderby=Eigenfactor).

#### Scopus CiteScore and SCimago Journal Rank (SJR) Indicators

Similar to WOS, Scopus is a large, multidisciplinary database provided by Dutch publisher, Elsevier, that covers a wide range of subject areas. CiteScore is part of the Scopus collection of research metrics that provides citation impact metrics for over 25,000 journals indexed in Scopus. The calculation of CiteScore metrics includes SJR (SCImago Journal Rank), SNIP (Source Normalized Impact per Paper), citation and document counts, and percentage cited. Both CiteScore and SJR use an algorithm similar to the Google Page Rank that orders the importance of websites by looking at the hyperlink structure of the World Wide Web.^17^–^19^

CiteScore does not rely on a two-year limit, but rather provides the average citation per document that a journal receives over a two-, three- and four-year period, with the additional analysis of SNIP that measures the impact of a paper within a subject field.^20^ Unlike JIF, CiteScore counts all documents in the denominator of the calculation, including editorials, letters, corrections, and case reports, which are less likely to be cited, and, therefore, lower the average metric score.^17^,^18^,^21^ The formula to calculate a three-year CiteScore for 2017 is illustrated below:

$$\text{Year }2017\;\text{CiteScore}=\frac{\text{Citations received to items published in }2014+2015+2016\;\text{Total counts of all documents}}{\text{published in }2014+2015+2016^{17}}$$

The metric data shown in Table 1 provides a brief analysis of the top 20 EM journals in 2017 JCR, Eigenfactor, SJR, and CiteScore. Four EM titles reported in JCR (*Adv Wound Care, Shock, Intern Emerg Med,* and *Crit Care Resusc*) were not grouped under the subject category of EM as in Scopus. Instead, the titles were categorized and ranked among other subject disciplines such as “Critical Care Medicine” and “Medicine, General.” To make a fair comparison, we placed and ranked these titles with the 26 EM journals in JCR and compared them with SJR and CiteScore. Among the top 20 EM journals found in SJR and CiteScore, three titles (*Curr* *Heart Fail Rep, West J Emerg Med* (*West*JEM), and *J Trauma Manag Outcomes*) are currently not indexed in SCIE, and only *West*JEM is indexed in the Emerging Sources Citation Index (ESCI), a new WOS database launched in 2015. See Table 1 to learn more about other ranking variations and findings among these metric indicators in EM journals.

#### Google Scholar: Journal-Level Metrics

Google Scholar metrics publishes the top 100 publications of the world’s journals every summer. The 2018 report (https://scholar.google.com/citations?view_op=top_venues) was released in August. The list is calculated using their five-year h-index and h-median metrics. The h-index has traditionally been used as an author-level metric, but in recent years it has been adapted to a journal-level metric by Google Scholar and SJR. The h-index of a journal is based on the set of most-cited articles published in that journal. It calculates the number (*h*) of most-cited papers published in that journal in the prior five years that were cited at least *h* times each. For example, *West*JEM received an h-5 index of 28 in the 2018 Scholar metric report. This means 28 papers published in the prior five years (from 2013 to 2017) in *West*JEM have been cited at least 28 times and was ranked #14 in the report. The h-5 index of the top 20 EM journals reported by Google Scholar in 2018 is at https://scholar.google.com/citations?view_op=top_venues&hl=en&vq=med_emergencymedicine.

### Author-Level Metrics: h-index

The h-index, developed by Hirsch, measures the total citations generated from an individual author’s publications based upon the most-cited articles.^22^ It expresses an author’s total number of papers (h) that have received at least ‘h’ citations. The h-index can easily be calculated manually by organizing an author’s articles in descending order of number of citations. As shown in Table 2, Author A published 10 papers that have been cited 40, 35, 28, 20, 15, 11, 9, 6, 5, and 2 times. The h-index in this case is seven because the seventh most-cited papers by this author have been cited at least seven times. When paper #8 receives two or more citations, the h-index will then move up to eight.

Commonly, junior faculty are penalized by the h-index. It takes years to build a body of publications and generate citations. Even with a few highly cited papers, a junior faculty member, in general, has fewer publications and citations than their senior colleagues. As shown in Table 2, Author B**,** who published three papers that were cited at least 15 times only generates an h-index of three. The h-index therefore cannot be used to compare a junior faculty member with a few publications and a senior faculty member with more years of publications and high citations.

Among academic emergency physicians, the h-index has been suggested as a way to “evaluate performance and identify emergency physicians with future success in EM research.”^23^,^24^ Both the author search function in WOS and Scopus can be used to create a report of an individual author’s overall citation counts, h-index, and publications. As with Google Scholar, individual authors can create a free scholar profile to track their publications and overall metric performance. Studies have found that Google Scholar yields a considerably larger number of “*Cited b*y” items than either WOS or Scopus,^25^ and nearly all academics had higher h-index in Google Scholar than in the two fee-based databases.^26^–^28^ Google Scholar yields broader and more comprehensive coverage for most disciplines from publishers, professional societies, and university repositories that allow access. Unlike WOS and Scopus, Google Scholar is free and provides unbiased retrievals of citations across disciplines. The reason that Google Scholar citations, and the corresponding h-indices, are higher than WOS or Scopus is that Google Scholar counts citations from all journals found on the web, while WOS and Scopus only count citations in a more restricted subset of journals that these indices include.

### Article-Level Metrics: Alternative Metrics

The journal- and citation-based metrics described above have limitations, which have been the subject of much criticism and debate in research and peer evaluation.^29^ They only measure a limited aspect of quality and no single metric can adequately reveal the full impact of research.^30^ In addition to the shortcomings of these traditional metric indicators, it takes years or decades to mature.^31^ Article-level metrics (ALMs) are an alternative approach to quantifying the research and impact of published research.

#### iCite

iCite is a metric web tool developed by the National Institutes for Health (NIH) for calculating Relative Citation Ratio (RCR) for PubMed articles. The purpose is to show the scientific influence of one or more articles relative to the average NIH-funded paper,^32^ and assess a researcher’s quality and productivity. The algorithm is based on an interconnected network of citations and uses a co-citation network to measure the impact of a paper within a subject field.^33^ The co-citation system enables comparison across scientific fields, e.g., comparing EM and critical care medicine. The article-level RCR is calculated by the total citations an article receives per year, divided by the average citations per year received by NIH-funded articles in the same field contemporaneously. Any article with RCR 1.0 has an RCR higher than 50% of NIH-funded papers, where 1.0 represents the field-normalized.^34^

The output data (e.g., total publications, publications per year, citations per year, RCR, and weighted RCR) produced by iCite can be used to understand the influence of articles within an analysis group. The NIH uses this application to determine the extent to which NIH awardees maintain high or low levels of influence in their respective fields of research.^32^ The figure illustrates a 2013 *West*JEM article, “*Oral and Intravenous Acetylcysteine for Treatment of Acetaminophen Toxicity: A Systematic Review and Meta-analysis.*” This paper’s mean RCR of 1.94 is higher than 73.8% of NIH-funded publications in EM.

As more scientists turn to social media and other “Web 2.0” platforms for communication and other scholarly activities, there is a need to measure the impact in non-traditional ways.^35^,^36^ These have led to the development of alternative metrics.^37^ “Altmetric” and other ALMs provide immediate measures and a more complete picture of the impact of scientific publications.^38^

#### Altmetric

Developed by Digital Science, Altmetric (https://www.altmetric.com/) is a web tracking system that measures impact by collecting relevant discussions and citations of each scholarly paper across the Internet and social media networks. These include peer reviews on Faculty of 1000 (http://f1000.com), citations on Wikipedia and in public policy documents, discussions on scientific blogs, mainstream news media coverage, bookmarks on reference managers (e.g., Mendeley), and mentions on social networks such as Facebook and Twitter.^39^

The Altmetric attention score is displayed with a colorful donut badge to help readers and researchers recognize the level and type of attention a paper receives in real time. At the time of completing this paper, an article published in *West*JEM in May 2016, “*Gender Differences in Emergency Department Visits and Detox Referrals for Illicit and Nonmedical Use of Opioids*” received an Altmetric score of 438. The article was mentioned by 54 news outlet, 11 tweeters, 1 Google+ user, and had eight Mendeley readers. In partnership with Altmetric, *West*JEM’s readers and authors can trace the real-time attention of this article at: https://escholarship.altmetric.com/details/9119550. Additionally, authors can view and track the top 10 *West*JEM articles mentioned recently in social media https://westjem.com/top-10-articles. As mentioned on its website, this added feature provides *West*JEM’s “authors with valuable feedback that gauges immediate impact of their work, long prior to article citation, the traditional metric of scholarly impact.”

Even in the era of alternative metrics, most research data remain uncited and the actual impact of alternative metrics in evaluating article impact remains uncertain.^40^ Conversely, a central criticism of alternative metrics is that they measure attention, and not necessarily quality.^40^ The most frequently shared or “newsworthy” papers might not be the most scientifically rigorous.^41^ A recent analysis of the top cited papers in EM suggested that there is a “mild correlation” between citation counts and Altmetric scores.^42^ Other studies have also shown that top cited articles can be predicted by the number of tweets about the article, especially in the first several days following publication.^43^

#### PlumX Metrics

PlumX, an article-level metric, recently acquired by Elsevier, offers authors an alternative approach to understand how their work is used and communicated online in near real time. Similar to Altmetric, PlumX metrics capture online activities associated with both general and academic audiences. Research resources include but are not limited to articles, conference proceedings, book chapters, and multimedia use. Using five major categories of metrics (“Usage,” “Captures,” “Mentions,” “Social Media,” and “Citations”), PlumX tracks citation activity that crosses traditional and alternative bibliometrics.^44^ After citation counts, the article-level usage metric is the next most-preferred metric among researchers.^45^ Authors can track their PlumX article-level metrics from a search result in Scopus^46^ and in EBSCOhost (EBSCO: Elton B. Stevens Company, a privately held company that provides online research services) databases.^47^

Lastly, a group of information professionals recently launched the Metrics Toolkit to assist researchers and scholars in navigating the ever-changing bibliometrics landscape. The site (http://www.metrics-toolkit.org/) provides links to the 27 most popular research measurement indicators for books, book chapters, datasets, journal articles, software, etc. It also includes an app that can recommend discipline-specific metrics to meet your needs. Best of all, the Metrics Toolkit carries a CC-BY 4.0 (Creative Commons Attribution 4.0 International) license so the content can be used at will.

### Strategies to Maximize Your Academic Success

#### Beware of Misleading Metrics and Fake Impact Factors

The bibliometrics described above are considered by the scientific community to be the measures of academic and scholarly productivity and scientific impact. Recently, the rise of so-called “predatory journals” has resulted in development of misleading, fake metrics that may fool novice researchers into believing that their works are being recognized and valued.^48^,^49^ Furthermore, predatory journals charge high article processing fees, but fail to provide the value of reputable publishers with legitimate peer review and wide indexing.^50^ They may advertise fabricated impact factors and other bibliometrics.^48^ Although there has not been research on the availability or use of these metrics, efforts have been made to identify and publicize these false metrics. These include the “Stop Predatory Journals” website https://predatoryjournals.com/metrics/ and a library subject guide that help researchers understand the significance and value of publishing in open access https://guides.lib.uci.edu/understanding_research_publishing.

To identify specific predatory journals to which you should avoid submission, go to https://predatoryjournals.com/journals/. In addition, you must also search in the predatory publishers list, as the predatory journals list only includes stand-alone journals, not those from multi-journal predatory publishers. Find these predatory publishers at https://predatoryjournals.com/publishers/. If neither the journal title nor publisher appears in either of these lists, the journal is likely legitimate.

#### Find the Right Journal for Your Research Paper

For inexperienced researchers, getting a research paper accepted for publication can be a challenge. To avoid rejections and delay in submission, it is crucial to choose the right journal. Here are the steps that can help you find journals that could be best suited for publishing your paper.

1. Conduct a literature search in PubMed to determine where related articles in your research topic have been published. Select the journals from the search results that match your research interests.
2. Check the journal’s indexing status in the NLM Catalog: Journals referenced in the NCBI Databases (https://www.ncbi.nlm.nih.gov/nlmcatalog/journals). Look for whether the journal is officially indexed in MEDLINE, PubMed, and PMC (PubMed Central). Avoid journals that are labeled as “*Only citations for author manuscripts are included,” “PubMed: Selected citations only.*” This indicates the least potential for visibility.
3. Go to the SJR Journal Ranking website and review the journal’s metrics, then query to further evaluate the specific ranking of the selected EM journals (http://www.scimagojr.com/journalrank.php?category=2711). Change the subject category at the top to assess rankings of journals in other fields.
4. After you identify the target journals that may match your paper and research, review the journal website to make sure that its scope and policies match your needs. In addition, check the journal’s review process and the instructions for authors thoroughly.
5. If you are still not sure, the tools shown in Table 3 can help to select the correct journals, as well as find relevant articles to cite in your manuscript. For journal editors, these tools can also help to identify potential reviewers.

In addition to the steps described above, we offered recommendations and key components of writing and publishing a successful research paper in our first article^2^ of this three-part series.

#### Consider non EM-specific Journals

With an exponential increase in the number of publications, particularly in widely-accessible open access journals, robust metrics that adequately describe the quality and impact of peer-reviewed publications is critical.^51^,^52^ In EM alone, there was a 58% increase in the number of specialty-based journals in the first decade of this century.^53^ The perceptions of EM as an academic specialty within the house of medicine are, in part, driven by how EM authors and reputable journals reach broader, non-EM audiences.^54^ It is important, therefore, to attempt to publish your work also in non-EM-specific journals. Some common examples are public health, healthcare management, critical care, ultrasound and disaster medicine, as well as traditional specialty journals outside of EM, such as cardiology, pediatrics, neurology, and toxicology.

#### Create a Google Scholar Profile to Track Research

Google Scholar offers a free and simple way to create a scholar profile that showcases your papers, calculates your h-index, and tracks citations. In addition, it can help you connect with scholars for potential future collaboration. Once you register and create a basic profile, Google Scholar provides you with a list of publications that may belong to you (with overlap of similar author surnames and initials). You validate your own publications and add them to your profile. After a profile is created, Google will automatically find and add your new publications. Other tracking features include the ability to see who is citing your publications, a graph of citations over time, and latest h- and i10-indices (articles cited at least 10 times).^55^ In addition, you can create email alerts to help you stay informed of new research in your area and to receive updates on new citations to your articles.

To gain more insight on promoting and bringing visibility to yourself and your scholarship, the second paper of this three-part series offers constructive guidance to junior faculty on strategies and resource tools such as creating an ORCID and engaging in social networks.^1^

## CONCLUSION

As an EM researcher’s career advances, the bibliometric tools and resources above should be considered when developing publication submission strategies. Publications in indexed, higher-impact journals are more likely to capture the impact and influence of scientific work performed by the EM researcher. The tenure and promotion decision process in many universities relies at least in part on these types of bibliometrics.^1^ Additionally, you now understand how newer, alternative metrics can be used to expand and promote scientific progress and your influence in new, more transparent, and timely ways.^38^

Finally, a word of wisdom from the authors: “The quality of your research and your contributions to the scientific community are of paramount importance. That brings the feeling of pride and honor, and is affected less by the prestige of the journal in which you publish.”

## Figures and Tables

**Table 1:** The comparison of top 20 emergency medicine journals in Journal Citation Report, Eigenfactor, SCImago Journal Rank (SJR), and CiteScore.

	JCR –top EM-related journals	JIF	Eigenfactor (ES) --Top EM-related journals	ES	SJR -- Top 20 EM journals	SJR	Scopus CiteScore – Top 20 EM journals	Citescore
1	Resuscitation	5.863	Resuscitation	0.02515	Resuscitation	2.643	Adv Wound Care^l^	6.21
2	Adv Wound Care^l^	5.2	Injury	0.01998	Ann Emerg Med	1.632	Resuscitation	3.81
3	Ann Emerg Med	4.680	Ann Emerg Med	0.01667	Acad Emerg Med	1.503	World J Emerg Surg	2.81
4	Emergencias^a^	3.608	Am J Emerg Med^b^	0.01478	Curr Heart Fail Rep^e^	1.468	Shock^j^	2.75
5	World J Emerg Surg	3.198	Acad Emerg Med	0.01354	Shock	1.331	Curr Heart Fail Rep^e^	2.73
6	Shock^j^	3.005	Shock^j^	0.01165	Prehosp Emerg Care	1.286	Injury	2.22
7	Acad Emerg Med	2.612	J of Emerg Med	0.01043	Adv Wound Care^l^	1.257	Prehosp Emerg Care	2.21
8	Intern Emerg Med^i^	2.453	Emerg Med J	0.00800	World J Emerg Surg	1.098	Acad Emerg Med	2.12
9	Scan J Trauma Resusc Emerg Med	2.312	Burns	0.00767	Burns	1.044	Burns	1.9
10	Prehosp Emerg Care	2.269	Ped Emerg Care	0.00655	Crit Care Resusc^f^	1.032	Scan J Trauma Resusc Emerg Med	1.7
11	Injury	2.199	Adv Wound Care^l^	0.00524	Injury	0.990	J Burn Care Res	1.57
12	Burns^j^	2.134	Scan J Trauma Resusc Emerg Med	0.00507	Emerg Med J	0.912	Ann Emerg Med	1.51
13	Emerg Med J	2.046	J Burn Care Res	0.00451	J Burn Care Res	0.768	Intern Emerg Med	1.48
14	Crit Care Resusc^f^	2.014	Intern Emerg Med	0.00433	Health Secur	0.739	Emerg Med Clin N Am	1.46
15	J Burn Care Res^k^	1.923	Prehosp Emerg Care	0.00375	Intern Emerg Med	0.735	Traumatology	1.43
16	Eur J Emerg Med^c^	1.729	Emerg Med Austr	0.00302	West J Emerg Med^g^	0.735	J Trauma Manag Outcomes^h^	1.42
17	Eur J Trauma Emerg Surg^d^	1.704	World J Emerg Surg	0.00276	Canad J Emerg Med	0.624	BMC Emerg Med	1.39
18	Canad J Emerg Med	1.481	Euro J Emerg Med^c^	0.00243	Emerg Med Austr	0.621	Emerg Med J	1.33
19	Emerg Med Clin N Am	1.429	Prehosp Disaster Med	0.00203	Scan J Trauma Resusc Emerg Med	0.618	Crit Care Resusc^f^	1.25
20	Emerg Med Austr	1.353	Euro J Trauma Emerg Surg^d^	0.00197	Am J Emerg Med^b^	0.604	West J Emerg Med^g^	1.24

a Emergencias was ranked #4 in 2017JCR, but was weighted much less by Eigenfactor (0.00116), SJR (0.603), and CiteScore (1.15).

b Am J Emerg Med was ranked #4 in Eigenfactor, but was weighted much lower in JCR (#21), SJR (#20), and CiteScore (#28).

c,d Both European journals are among the top 20 in JCR and Eignefactor, but that is not the case with SJR nor CiteScore..

e,g,h These journals were ranked among the top EM journals in SJR and CiteScore, but none are indexed in SCI Expanded Collection. Only WestJEM is indexed in WOS ESCI.

hˆ was ranked #34 in SJR, but ranked #16 in CiteScore.

f, i, j, k, l These journals were not categorized among the 26 emergency medicine journals found in JCR. Instead, they were grouped under other medical subject disciplines, e.g., “Critical Care Medicine.”

JIF, journal impact factor; ES, Eigenfactor Score.

**Table 2:** The calculation of h-index of an individual author’s publications.

Publications	Paper #1	Paper #2	Paper #3	Paper #4	Paper #5	Paper #6	Paper #7	Paper #8	Paper #9	Paper #10	h-Index
Author A											
Cited by	40	35	28	20	15	11	9	6	5	2	**7**
Author B											
Cited by	40	30	15								**3**

**Table 3:** Publishing tools to identify promising journals to which to submit your research paper.

Tool and weblink	Description
Jane (Journal/Author Name Estimator http://jane.biosemantics.org/	This website compares your abstract to millions of documents in PubMed. The results offer the best matching journals for your paper.
About Edanz https://www.edanzediting.com/about Edanz Journal Selection https://www.edanzediting.com/services/journal-selection	A fee-based editing service that is designed to help non-native English researchers to publish in international journals. Offers a list of three target journals that best match your research topic. Registration is required.
Elsevier Find a Journal https://www.elsevier.com/authors/journal-authors/submit-your-paper#find Match your Manuscript -- “Find the perfect journal for your article” https://journalfinder.elsevier.com	Search an Elsevier journal by name or enter your abstract in the “Match Your Manuscript” journal finder to locate potential Elsevier journals that are most suited for your research.
PubMed PubReMiner http://hgserver2.amc.nl/cgi-bin/miner/miner2.cgi	Allows you to run a search to determine journals that published the most articles relating to your topic.
Springer Journal Suggester https://journalsuggester.springer.com/	Enter your abstract, description of your research, or a sample text. The results will return with a list of relevant Springer and BioMed Central journals that are most suited for your research.
